# Comparison of Six Different Percutaneous Coronary Intervention Guidance Modalities

**DOI:** 10.3390/jcdd9100343

**Published:** 2022-10-08

**Authors:** Mengjin Hu, Jiangshan Tan, Yuejin Yang

**Affiliations:** State Key Laboratory of Cardiovascular Disease, Fuwai Hospital, National Center for Cardiovascular Diseases, Chinese Academy of Medical Sciences & Peking Union Medical College, Beijing 100037, China

**Keywords:** percutaneous coronary intervention, coronary angiography, intravascular ultrasound, optical coherence tomography, fractional flow reserve

## Abstract

Background: New randomized trials and modalities in guiding percutaneous coronary intervention (PCI) have become available. Objective: We aimed to compare the clinical outcomes of coronary angiography (CAG), intravascular ultrasound (IVUS), optical coherence tomography (OCT), fractional flow reserve (FFR), instantaneous wave-free ratio (iFR), and optical frequency domain imaging (OFDI)-guided PCI. Methods: A network meta-analysis was performed to compare different PCI guidance modalities. The clinical outcomes included major adverse cardiovascular events (MACE), all-cause death, myocardial infarction (MI), and target vessel/lesion revascularization (TVR/TLR). Odds ratio (OR) and corresponding 95% credible interval (CrI) were calculated. Results: Thirty-six randomized trials, including 19,042 patients, were included. In comparison with CAG, IVUS significantly reduced MACE (OR: 0.71; 95% CrI: 0.57 to 0.86) and TVR/TLR (OR: 0.53; 95% CrI: 0.43 to 0.66). MACE (OR: 1.44; 95% CrI: 1.02 to 2.08) and TVR/TLR (OR: 1.87; 95% CrI: 1.04 to 3.71) were significantly increased in the FFR group, compared with IVUS group. There were no significant differences in MACE or TVR/TLR among the left guidance modality comparisons. Differences in all-cause death or MI were not observed in any comparisons. Conclusions: IVUS could reduce MACE and TVR/TLR, compared with CAG or FFR. Therefore, IVUS may be the optimal modality in guiding PCI.

## 1. Introduction

Coronary angiography (CAG) remains the most widely used percutaneous coronary intervention (PCI) guidance modality. However, CAG has numerous inherent limitations, including two-dimensional projection to define the structure of complex three-dimensional coronary artery lumens and limited ability in assessing the vessel wall, plaque composition, and degree of atherosclerosis [1]. The later-developed intravascular ultrasound (IVUS) and optical coherence tomography (OCT) can provide detailed visualization of intraluminal and transmural coronary anatomy, overcoming many limitations inherent in CAG [2]. Physiological measurements obtained using fractional flow reserve (FFR) have also been used to guide PCI, which can provide additional information complementary to CAG. Instantaneous wave-free ratio (iFR) is a pressure-derived index of stenosis severity similar to FFR but without the use of adenosine. Among patients with stable angina or acute coronary syndrome (ACS), iFR-guided PCI was noninferior to FFR-guided PCI concerning the 12-month major adverse cardiovascular events (MACE) rate [3]. Optical frequency domain imaging (OFDI) is a newly developed second-generation OCT, which enables the three-dimensional reconstruction of complex anatomies and their relationship with the metallic structure [4]. The OPTIMUM trial (Online 3-Dimensional Optical Frequency Domain Imaging to Optimize Bifurcation Stenting Using UltiMaster Stent) demonstrated that OFDI-guided PCI could reduce the risk of acute incomplete stent apposition at the bifurcation, compared with CAG-guided PCI [3].

Although numerous modalities have become available in guiding PCI, until now, no meta-analysis has been published to compare the clinical outcomes of all available modalities (CAG, IVUS, OCT, OFDI, FFR, iFR) within a single analytical framework. Therefore, in this network meta-analysis, we sought to systematically review randomized trials that assessed the clinical effects of different PCI guidance modalities on patients with coronary artery disease.

## 2. Methods

The network meta-analysis complies with the PRISMA (preferred reporting items for systematic reviews and meta-analyses) network meta-analysis extension statement [5]. It has been registered at the PROSPERO international prospective register of systematic reviews (CRD42021284232).

### 2.1. Search Strategy

Cochrane Central Register of Controlled Trials (CENTRAL), MEDLINE, EMBASE, Web of Science, TCTMD, ClinicalTrials.gov, and major congress proceedings from inception date to 8 March 2022 were searched to identify potential studies, using combinations of the medical subject headings or keywords “coronary angiography”, “CAG”, “intravascular ultrasound” “IVUS”, “optical coherence tomography”, “OCT”, “fractional flow reserve”, “FFR”, “instantaneous wave-free ratio”, “iFR”, “optical frequency domain imaging”, “OFDI”, “percutaneous coronary intervention”, “PCI”, “randomized controlled trial”, “randomized trial”, “trial”. To supplement the search of the electronic databases, we also searched relevant randomized trials from reference lists of identified systematic reviews, meta-analyses, and relevant reviews.

### 2.2. Selection Criteria and Data Extraction

Randomized trials that compared the following PCI guidance modalities (CAG, IVUS, OCT, FFR, iFR, OFDI) in patients with coronary artery disease were included. Studies were excluded if they did not report interesting clinical outcomes. In the case of multiple publications from the same randomized trial, we included the publication with the most extended follow-up clinical outcomes. For missing data, we contacted authors directly or retrieved data from relevant systematic reviews and meta-analyses. Two independent reviewers (M.H. and J.T.) conducted the processes of selection and data extraction. Disagreement was resolved through consensus with a third-party reviewer (Y.Y.).

### 2.3. Quality Assessment of the Risk of Bias

We assessed the risk of bias by the Cochrane risk of bias assessment tool [6], which includes the assessment of the following items: random sequence generation, allocation concealment, blinding of participants and personnel, blinding of outcome assessment, incomplete outcome data, selective reporting, and other bias. We investigated publication bias with comparison-adjusted funnel plots.

### 2.4. Clinical Outcomes

MACE, all-cause death, myocardial infarction (MI), and target vessel/lesion revascularization (TVR/TLR) were analyzed separately. The MACE was defined according to per individual trial.

### 2.5. Statistical Analysis

A hierarchical Bayesian network meta-analysis was performed to estimate the odds ratio (OR) with 95% credible interval (CrI) under random effects consistency models. We also calculated the surface under the cumulative ranking curve (SUCRA) to compare the relative ranking probability of each modality [7]. We statistically evaluated consistency with the “loop specific” approach, and then separated direct evidence from indirect evidence using node splitting. Heterogeneity was interpreted by the I^2^ statistic, with values of <25%, 25–50%, and >50% representing low, moderate, and high degrees of heterogeneity [8]. Three Markov chains were run at the same time with 100,000 simulated draws after a burn-in of 50,000 iterations. Publication bias was evaluated by visual inspection of funnel plots. Funnel plots were drawn by Stata 14 SE (StataCorp, College Station, TX, USA). The left statistical evaluations were conducted using the “gemtc” package from R software (version 3.4.3). 

## 3. Results

### 3.1. Characteristics of Included Studies

**Figure 1:**
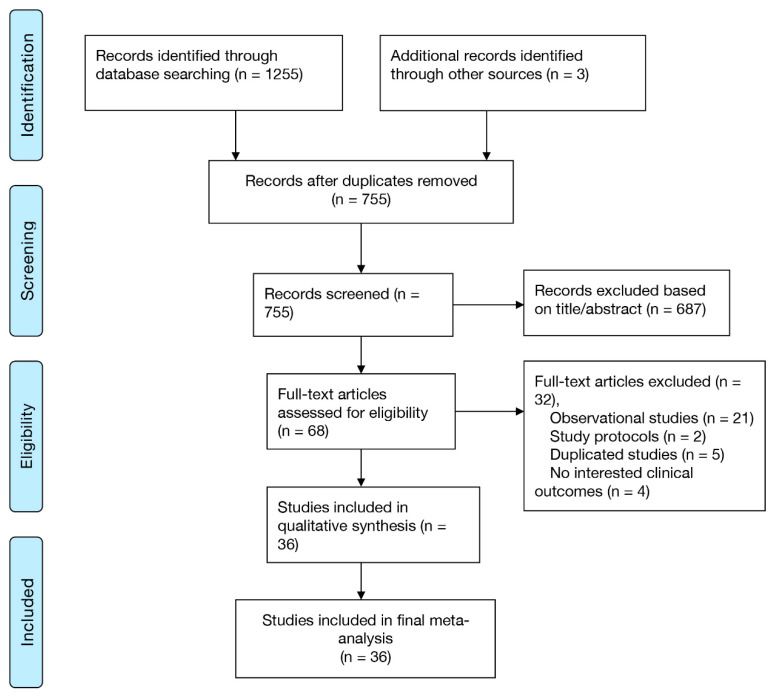
PRISMA diagram for study inclusion. CAG: coronary angiography; FFR: fractional flow reserve; iFR: instantaneous wave-free ratio; IVUS: intravascular ultrasound; OCT: optical coherence tomography; OFDI: optical frequency domain imaging.

As depicted in Figure 1, our electronic search identified 1255 potentially relevant studies, three records were identified through other sources. Eventually, thirty-six randomized trials reporting on 19,042 patients were included. Eighteen studies were comparisons between IVUS and CAG [9,10,11,12,13,14,15,16,17,18,19,20,21,22,23,24,25,26], three were comparisons between OCT and CAG [27,28,29], one was a comparison between OFDI and CAG [4], one was a comparison between OCT and IVUS [30], seven were comparisons between FFR and CAG [31,32,33,34,35,36,37], two were comparisons between OFDI and IVUS [38,39], one was a comparison between OCT and FFR [40], two were comparisons between FFR and iFR [3,41], one was a comparison among OCT, IVUS, and CAG [42]. The number of patients in the CAG, IVUS, OCT, FFR, iFR, and OFDI groups were 6501, 5014, 611, 4126, 2268, and 522, respectively. The network evidence plot is shown in Figure S1. The included randomized trials were overall moderate, with only some studies revealing a high risk of bias (Figure S2). Supplementary Tables S1 and S2 summarize the characteristics of included studies and patients, respectively.

### 3.2. MACE

**Figure 2:**
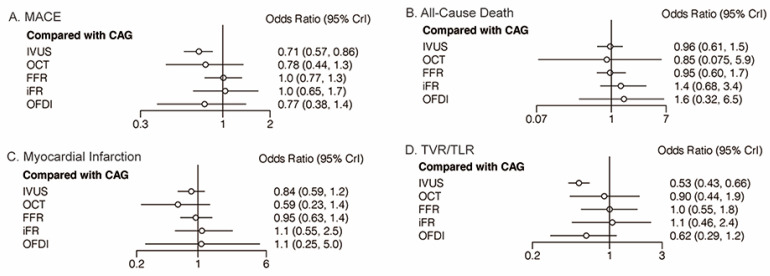
Comparisons of clinical outcomes among guidance modalities included in the network meta-analysis. CAG: coronary angiography; FFR: fractional flow reserve; iFR: instantaneous wave-free ratio; IVUS: intravascular ultrasound; MACE: major adverse cardiovascular events; OCT: optical coherence tomography; OFDI: optical frequency domain imaging; TVR/TLR: target vessel/lesion revascularization. (**A**) MACE; (**B**) All-Cause Death; (**C**) Myocardial Infarction; (**D**) TVR/TLR.

**Figure 3:**
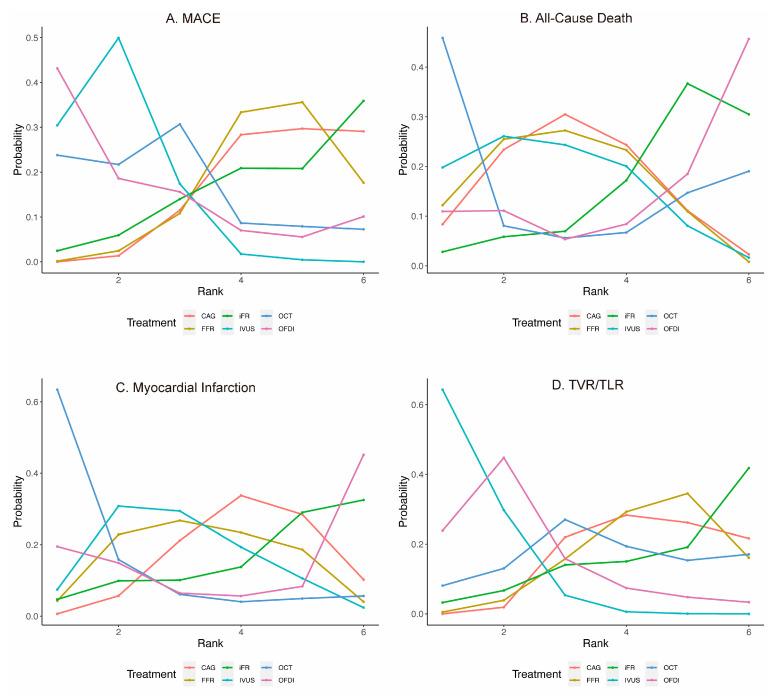
Rank probability analysis for clinical outcomes among guidance modalities included in the network meta-analysis. CAG: coronary angiography; FFR: fractional flow reserve; iFR: instantaneous wave-free ratio; IVUS: intravascular ultrasound; MACE: major adverse cardiovascular events; OCT: optical coherence tomography; OFDI: optical frequency domain imaging; TVR/TLR: target vessel/lesion revascularization. (**A**) MACE; (**B**) All-Cause Death; (**C**) Myocardial Infarction; (**D**) TVR/TLR.

Twenty-eight trials (17,436 patients) reported 1580 (9.06%) MACE events. As shown in Figure 2A, IVUS could reduce the risk of MACE in comparison with CAG (OR: 0.71; 95% CrI: 0.57 to 0.86). FFR was also associated with a higher risk of MACE, compared with IVUS (OR: 1.44; 95% CrI: 1.02 to 2.08). No significant differences existed in the remaining comparisons (Table 1). Direct pairwise comparison analyses yielded similar results to network analyses (Figure S3). Figure 3A demonstrates the calculated SUCRA and mean rank for MACE by the modality options. Generally, OFDI had the highest probability for reducing MACE, followed by IVUS (Figure 3A).

### 3.3. All-Cause Death

Thirty trials (15935 patients) reported 326 (2.05%) cases of all-cause death. Compared with CAG, no significant differences in all-cause death existed with IVUS, OCT, FFR, iFR, or OFDI (Figure 2B). Similarly, we found no significant differences in the remaining comparisons (Table 1). In the pairwise analyses, similar results were observed (Figure S4). OCT had the highest probability for reducing all-cause death (Figure 3B).

### 3.4. MI

Thirty-three trials (18,460 patients) reported 556 (3.01%) MI events. Compared with CAG, no significant differences in MI were observed with IVUS, OCT, FFR, iFR, or OFDI (Figure 2C). Similarly, no significant differences existed in the remaining comparisons (Table 1). Similar results were obtained with the pairwise meta-analysis (Figure S5). OCT had the highest probability for reducing MI (Figure 3C). 

### 3.5. TVR/TLR

Twenty-eight trials (12,937 patients) reported 639 (4.94%) TVR/TLR events. IVUS could reduce the risk of TVR/TLR, compared with CAG (OR: 0.53; 95% CrI: 0.43 to 0.66), whereas OCT, FFR, iFR, or OFDI could not (Figure 2D). Moreover, FFR was associated with a higher risk of TVR/TLR relative to IVUS (OR: 1.87; 95% CrI: 1.04 to 3.71) (Table 1). In the pairwise analyses, similar results were observed (Figure S6). IVUS had the highest probability for reducing TVR/TLR (Figure 3D). 

### 3.6. Publication Bias and Heterogeneity

No publication bias was observed for MACE (Figure S7A), all-cause death (Figure S7B), MI (Figure S7C), or TVR/TLR (Figure S7D). Heterogeneity was also low among the examined outcomes.

### 3.7. Sensitivity Analyses

After leaving out the studies using bare metal stent or conducted before 2000, IVUS consistently showed benefits in MACE (Figure S8A), MI (Figure S8C), and TVR/TLR (Figure S8D). In patients with acute coronary syndrome, IVUS showed benefits in decreasing MACE (Figure S9A), all-cause death (Figure S9B), MI (Figure S9C), and TVR/TLR (Figure S9D). However, no benefits were found in patients with stable angina (Figure S10). In patients with complex lesions, IVUS could decrease MACE (Figure S11A) and MI (Figure S11C). In patients without complex lesions, a trend towards decreased risks of MACE, all-cause death, MI, and TVR/TLR was observed with IVUS (Figure S12).

## 4. Discussion

The present network meta-analysis of 36 randomized trials with 19,042 patients revealed the lower risks of MACE and TVR/TLR associated with IVUS, compared with CAG or FFR. No differences in all-cause death or MI were observed in any comparisons. These results suggested that IVUS may be the optimal modality in guiding PCI.

IVUS is helpful in providing information on lesion characteristics, such as vulnerable plaques, lesion severity, length, and morphology [22]. Moreover, IVUS can give information on stent status, which can translate into optimal stent expansion to overcome the potentially harmful effects, especially in longer drug-eluting stents (≥28 mm) [18]. Previous meta-analyses have mainly focused on the comparison between CAG and IVUS. With the emergence of more PCI guidance modalities, it is unknown whether IVUS is better than these newly developed PCI guidance modalities. Our network meta-analysis suggested that IVUS was superior to CAG in reducing MACE and TVR/TLR. A similar scenario was also observed for the comparison between IVUS and FFR.

In another Bayesian network meta-analysis, including 31 studies (17 randomized trials and 14 propensity score weight-matched studies) and 17882 patients, clinical outcomes of CAG, IVUS, or OCT/OFDI-guided PCI were compared. The study demonstrated that IVUS could significantly reduce the risks of MACE (OR: 0.79; 95% CrI: 0.67 to 0.91), all-cause death (OR: 0.74; 95% CrI: 0.58 to 0.98), cardiovascular death (OR: 0.47; 95% CrI: 0.32 to 0.66), MI (OR: 0.72; 95% CrI: 0.52 to 0.93), TLR (OR: 0.74; 95% CrI: 0.58 to 0.90), and stent thrombosis (OR: 0.42; 95% CrI: 0.20 to 0.72), compared with CAG. OCT/OFDI was associated with a significant reduction in MACE (OR: 0.68; 95% CrI: 0.49 to 0.97) and cardiovascular death (OR: 0.31; 95% CrI: 0.13 to 0.66), compared with CAG. No differences existed between IVUS and OCT/OFDI. In a subgroup analysis just based on randomized trials, the effect of IVUS on all-cause death disappeared [43], which means that more randomized trials may be needed to demonstrate the effect of IVUS on all-cause death. In another network meta-analysis conducted by Iannaccone et al., a total of 33 studies (16 randomized trials and 17 propensity score weight-matched studies) comparing IVUS, FFR, OCT, and CAG-guided PCI were included. IVUS could reduce the risk of MACE, compared with CAG (OR: 0.75; 95% CrI: 0.52 to 0.88), which was mainly driven by reduced risks of all-cause death (OR: 0.75; 95% CrI: 0.50 to 0.97), MI (OR: 0.82; 95% CrI: 0.54 to 0.94), stent thrombosis (OR: 0.78; 95% CrI: 0.60 to 0.98), and revascularization. FFR also reduced all-cause death (OR: 0.78; 95% CrI: 0.63 to 0.98), MI (OR: 0.74; 95% CrI:0.57 to 0.99), and revascularization, compared with CAG [44]. It is noteworthy that in the aforementioned network meta-analyses, the number of randomized trials was limited and observational studies were also included, which may introduce bias and limit the power to detect real differences. In our network meta-analysis, a total of 36 randomized trials were included, while observational studies were excluded, which enables a relatively high-quality assessment of available PCI guidance modalities. We found that IVUS-guided PCI could reduce the risks of MACE and TVR/TLR, compared with CAG or FFR, indicating that IVUS may be the optimal modality in guiding PCI.

However, despite accumulating randomized trials and meta-analysis [21,22,23,25,26,45,46] supporting the use of IVUS to optimize PCI, in real-world scenarios, the adoption of IVUS in guiding PCI remains low. In an extensive database from the United States, 3,211,872 hospitalizations were included between 2004 and 2014. IVUS was performed just in 87,804 cases (2.7%). Fortunately, the percentage of intravascular imaging use was increased with time going on, from 2.1% in 2004–2005 to 6.6% in 2013–2014 (P trend < 0.001) [47]. Meanwhile, the limitations associated with IVUS should also be considered. After all, IVUS-guided PCI required longer fluoroscopy time and procedural time, compared with CAG-guided PCI, [9,11,16] which resulted in a subsequent increased level of contrast volume [16].

Similar to IVUS, OCT is another intravascular imaging modality with higher resolution, which can evaluate the surface vascular changes and stent strut coverage. However, the lower penetration depth (1 to 2 mm) of OCT may make assessment of plaque volume or visualization of plaques in the deep layers of the vessel wall unfeasible. 

Several limitations deserve attention. First, the indications for PCI, definitions of MACE, duration of follow-up, and patient characteristics were heterogeneous across the randomized trials, which may add heterogeneity and limit the extrapolation of our study. However, heterogeneity was low among the examined outcomes. Second, due to the limited number of randomized trials, the 95% CrI for stent thrombosis was wide (Figure S13). Third, a longer learning curve is required to command a new PCI guidance modality. Therefore, we cannot exclude the possibility that, in the early application stage of a new modality, being unfamiliar with it may negatively affect prognosis.

## 5. Conclusions

IVUS-guided PCI could reduce the risks of MACE and TVR/TLR, compared with CAG and FFR. No differences in all-cause death or MI were observed in any comparisons. Therefore, IVUS may be the optimal modality in guiding PCI. Moreover, with the advent of more PCI guidance modalities, further research is required to validate their application in clinical practice.

## Figures and Tables

**Table 1:** Main analysis for clinical outcomes.

Outcomes	OR (95% CrI)	OR (95% CrI)	OR (95% CrI)	OR (95% CrI)	OR (95% CrI)
MACE					
CAG	0.71 (0.57, 0.86)	0.78 (0.44, 1.30)	1.00 (0.77, 1.30)	1.00 (0.65, 1.70)	0.77 (0.38, 1.40)
	IVUS	1.09 (0.63, 2.05)	1.44 (1.02, 2.08)	1.44 (0.88, 2.52)	1.05 (0.51, 1.97)
		OCT	1.31 (0.75, 2.21)	1.34 (0.68, 2.51)	0.93 (0.38, 2.19)
			FFR	1.01 (0.68, 1.49)	0.73 (0.33, 1.49)
				iFR	0.72 (0.3, 1.61)
					OFDI
All-Cause Death					
CAG	0.96 (0.61, 1.50)	0.85 (0.08, 5.90)	0.95 (0.60, 1.70)	1.40 (0.68, 3.40)	1.60 (0.32, 6.50)
	IVUS	0.92 (0.14, 7)	1.06 (0.57, 2.12)	1.59 (0.65, 4.16)	1.79 (0.43, 7.60)
		OCT	1.16 (0.17, 7.60)	1.70 (0.22, 12.40)	1.77 (0.21, 17.79)
			FFR	1.48 (0.77, 2.91)	1.70 (0.34, 8.29)
				iFR	1.15 (0.20, 6.09)
					OFDI
Myocardial Infarction					
CAG	0.84 (0.59, 1.20)	0.59 (0.23, 1.40)	0.95 (0.63, 1.40)	1.10 (0.55, 2.50)	1.10 (0.25, 5.00)
	IVUS	0.67 (0.26, 1.85)	1.06 (0.68, 2.01)	1.27 (0.61, 3.19)	1.26 (0.31, 6.14)
		OCT	1.61 (0.58, 4.54)	1.92 (0.60, 6.23)	1.95 (0.33, 10.67)
			FFR	1.21 (0.66, 2.13)	1.20 (0.26, 5.41)
				iFR	1.00 (0.18, 5.26)
					OFDI
TVR/TLR					
CAG	0.53 (0.43, 0.66)	0.90 (0.44, 1.90)	1.00 (0.55, 1.80)	1.10 (0.46, 2.40)	0.62 (0.29, 1.20)
	IVUS	1.69 (0.73, 3.44)	1.87 (1.04, 3.71)	2.06 (0.85, 5.13)	1.19 (0.67, 2.16)
		OCT	1.14 (0.57, 2.67)	1.27 (0.47, 3.43)	0.72 (0.29, 2.07)
			FFR	1.08 (0.60, 1.92)	0.63 (0.27, 1.52)
				iFR	0.58 (0.21, 1.69)
					OFDI

CAG: coronary angiography; FFR: fractional flow reserve; iFR: instantaneous wave-free ratio; IVUS: intravenous ultrasound; MACE: major adverse cardiovascular events; OCT: optical coherence tomography; OFDI: optical frequency domain imaging; TVR/TLR: target vessel/lesion revascularization.

## Data Availability

The datasets generated during and/or analyzed during the current study are not publicly available but are available from the corresponding author on reasonable request.

## Supplementary Materials

The following supporting information can be downloaded at: https://www.mdpi.com/article/10.3390/jcdd9100343/s1, Figure S1: Different Guidance Modalities Included in the Network Meta-Analysis; Figure S2: Quality Assessment Summary of Included Studies; Figure S3: Major Adverse Cardiovascular Events of Network Node-Split; Figure S4: All-Cause Death of Network Node-Split; Figure S5: Myocardial Infarction of Network Node-Split; Figure S6: Target Vessel/Lesion Revascularization of Network Node-Split; Figure S7: Funnel Plot of Publication Bias for Major Adverse Cardiovascular Events (A), All-Cause Death (B), Myocardial Infarction (C), and Target Vessel/Lesion Revascularization (D); Figure S8: Sensitivity Analysis Leaving Out Studies Using Bare Metal Stent or Conducted before 2000; Figure S9: Sensitivity Analysis Focusing on Patients with Acute Coronary Syndrome; Figure S10: Sensitivity Analysis Focusing on Patients with Stable Angina; Figure S11: Sensitivity Analysis Focusing on Patients with Complex Lesions; Figure S12: Sensitivity Analysis Focusing on Patients without Complex Lesions; Figure S13: Comparisons of Stent Thrombosis Among Guidance Modalities Included in the Network Meta-Analysis; Table S1: Characteristics of Studies Included in the Network Meta-Analysis; Table S2: Clinical Characteristics of Patients Across Studies Included in the Network Meta-Analysis. Refs. [48,49,50,51] are cited in the Supplementary Materials.
