# Supplementary material for: Comparison of Six Different Percutaneous Coronary Intervention Guidance Modalities
Source: J Cardiovasc Dev Dis. 2022 Oct 8;9(10):343. doi: 10.3390/jcdd9100343 (PMC9604203; doi:10.3390/jcdd9100343)

Figure S1 Different Guidance Modalities Included in the Network Meta-Analysis (A)

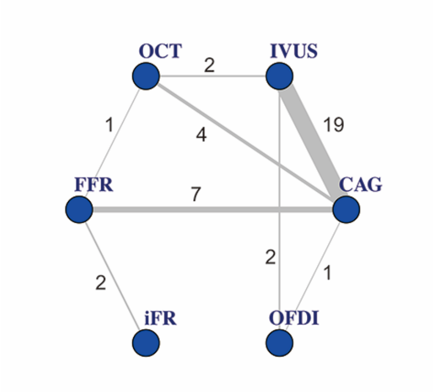

CAG: coronary angiography; FFR: fractional flow reserve; iFR: instantaneous wave-free ratio; IVUS: intravascular ultrasound; OCT: optical coherence tomography; OFDI: optical frequency domain imaging

Figure S2 Quality Assessment Summary of Included Studies

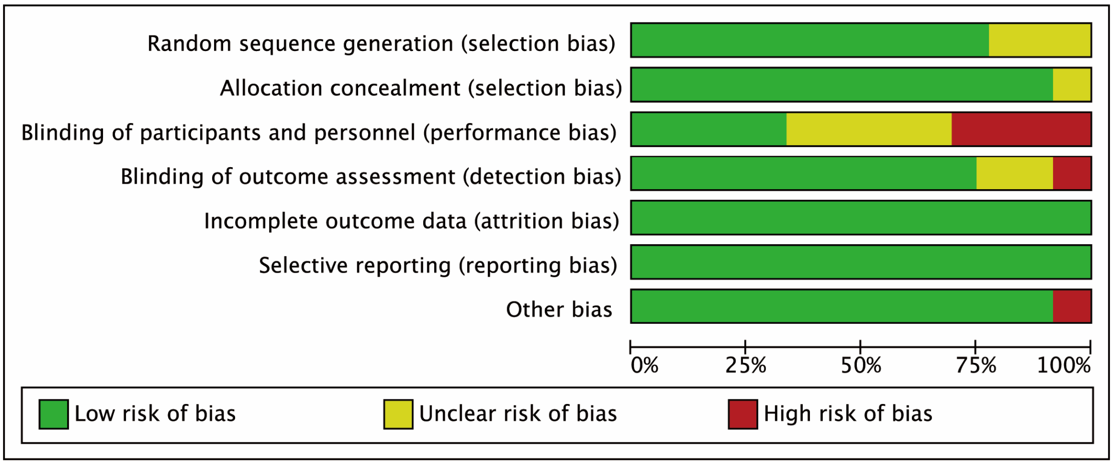

CAG: coronary angiography; FFR: fractional flow reserve; iFR: instantaneous wave-free ratio; IVUS: intravascular ultrasound; OCT: optical coherence tomography; OFDI: optical frequency domain imaging

Figure S3 Major Adverse Cardiovascular Events of Network Node-Split

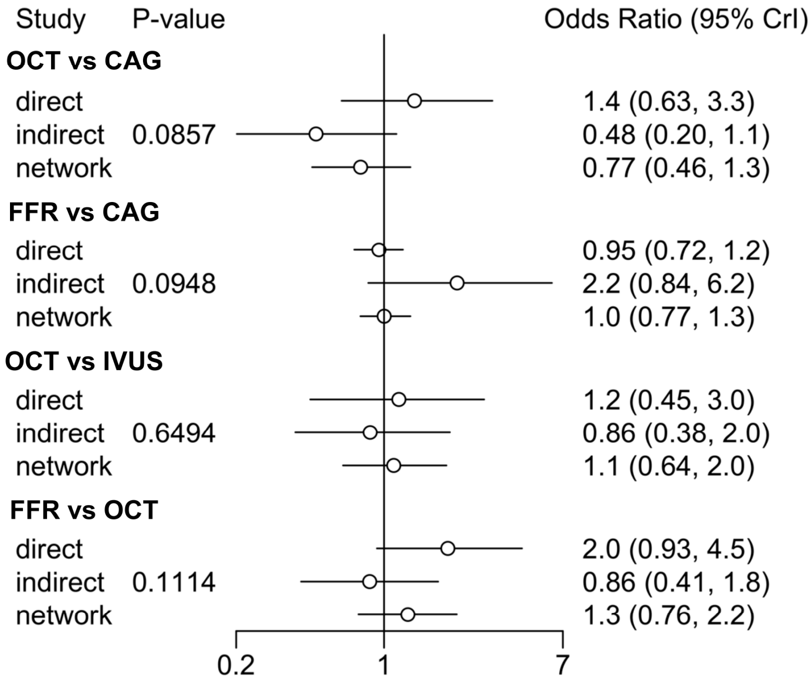

CAG: coronary angiography; FFR: fractional flow reserve; IVUS: intravascular ultrasound; OCT: optical coherence tomography

Figure S4 All-Cause Death of Network Node-Split

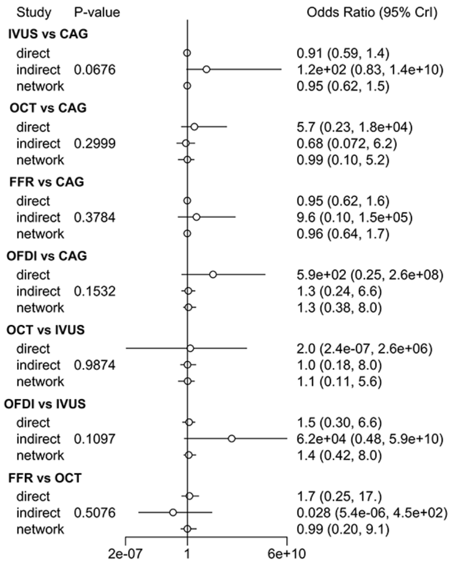

CAG: coronary angiography; FFR: fractional flow reserve; IVUS: intravascular ultrasound; OCT: optical coherence tomography; OFDI: optical frequency domain imaging

Figure S5 Myocardial Infarction of Network Node-Split

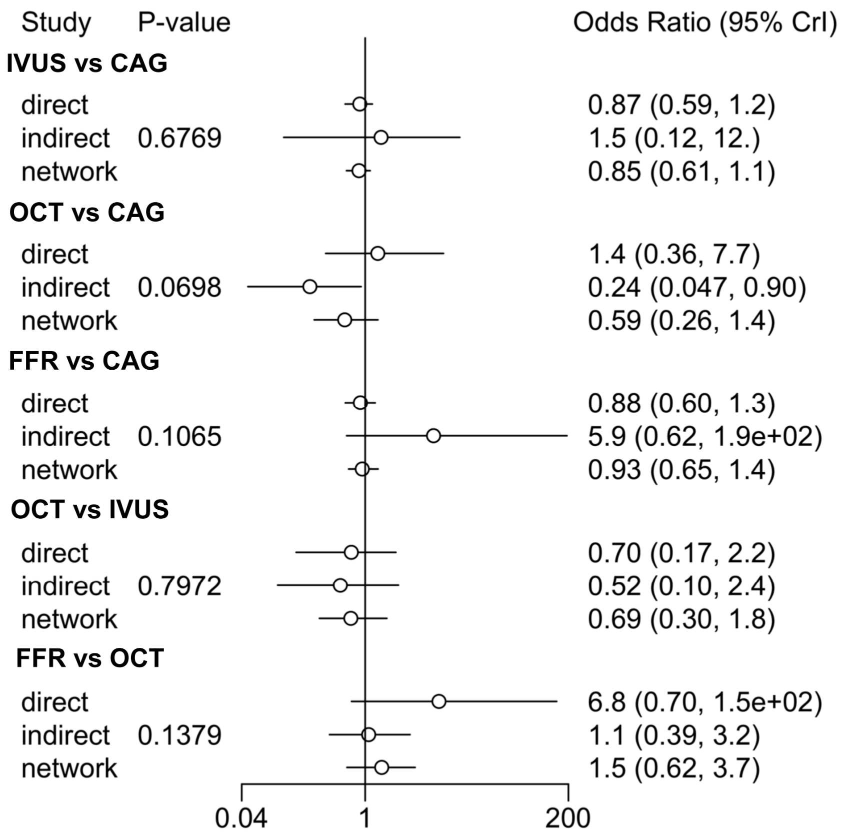

CAG: coronary angiography; FFR: fractional flow reserve; IVUS: intravascular ultrasound; OCT: optical coherence tomography

Figure S6 Target Vessel/Lesion Revascularization of Network Node-Split

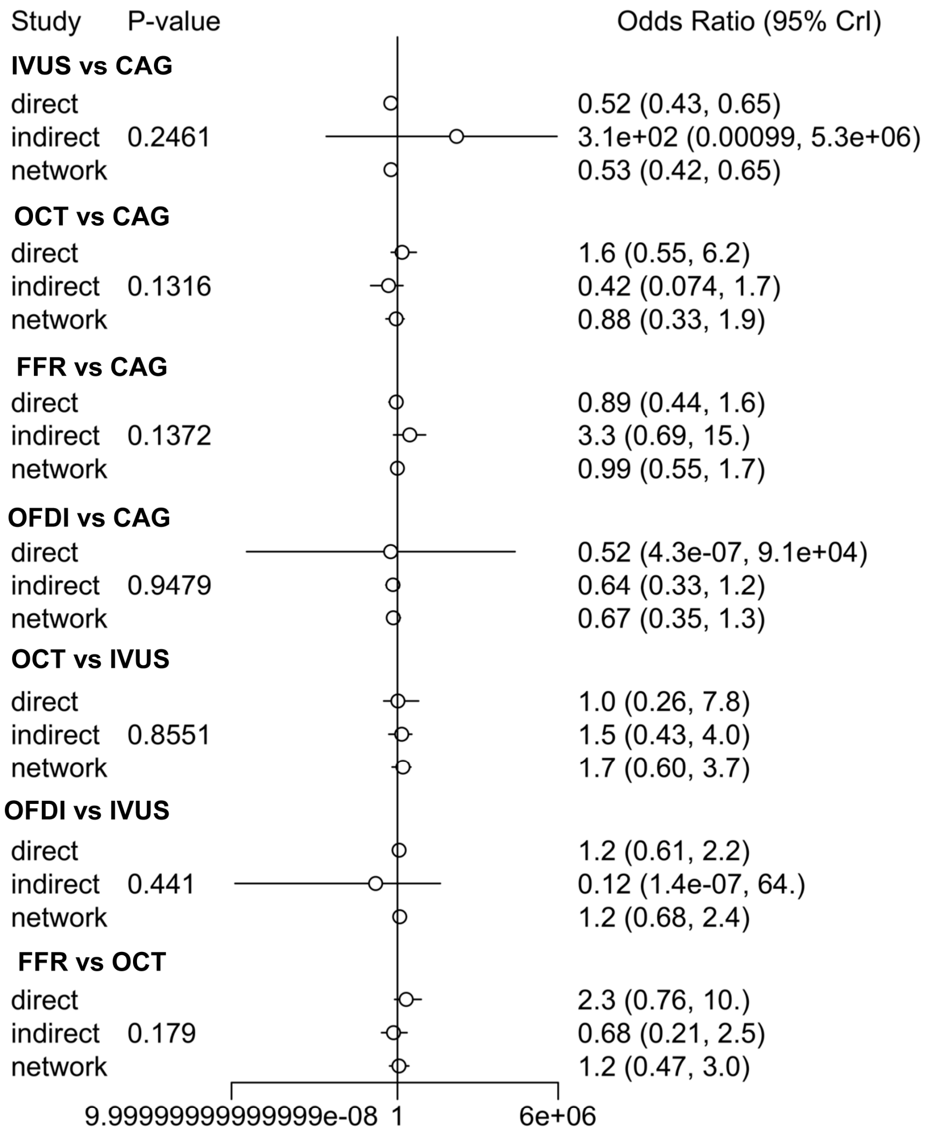

CAG: coronary angiography; FFR: fractional flow reserve; IVUS: intravascular ultrasound; OCT: optical coherence tomography; OFDI: optical frequency domain imaging

Figure S7 Funnel Plot of Publication Bias for Major Adverse Cardiovascular Events (A), All-Cause Death (B), Myocardial Infarction (C), and Target Vessel/Lesion Revascularization (D)

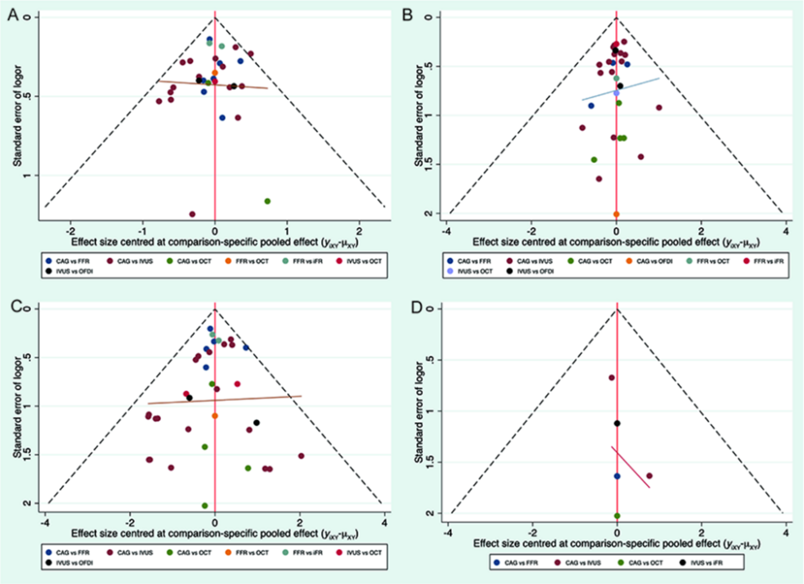

CAG: coronary angiography; FFR: fractional flow reserve; iFR: instantaneous wave-free ratio; IVUS: intravascular ultrasound; OCT: optical coherence tomography; OFDI: optical frequency domain imaging

Figure S8 Sensitivity Analysis Leaving Out Studies Using Bare Metal Stent or Conducted before 2000

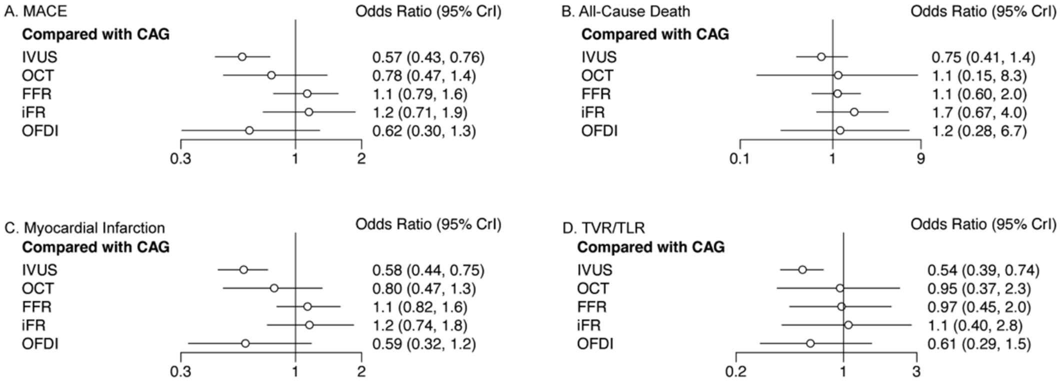

Figure S9 Sensitivity Analysis Focusing on Patients with Acute Coronary Syndrome

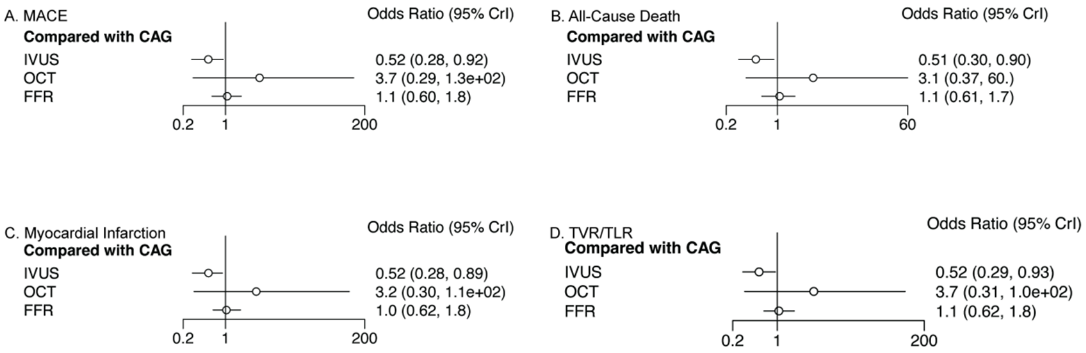

Figure S10 Sensitivity Analysis Focusing on Patients with Stable Angina

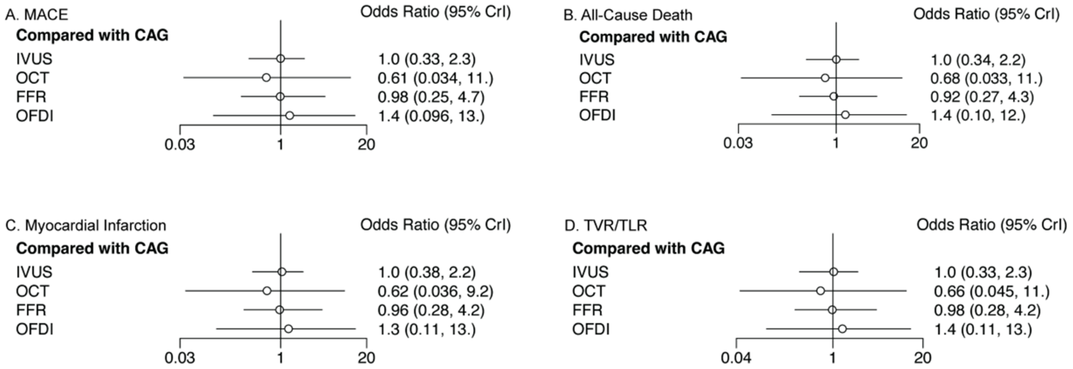

Figure S11 Sensitivity Analysis Focusing on Patients with Complex Lesions

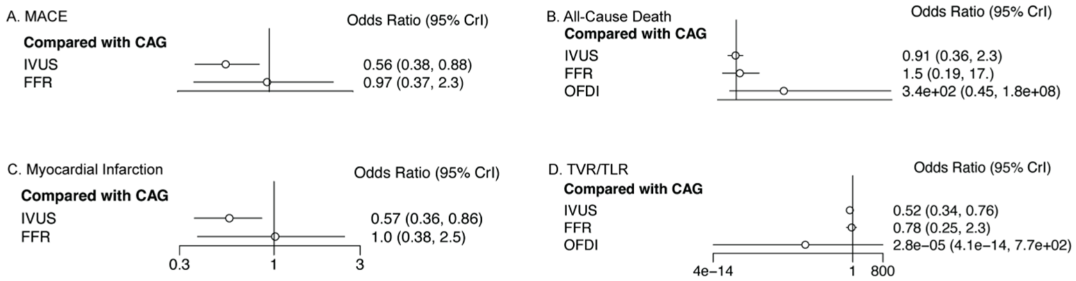

Figure S12 Sensitivity Analysis Focusing on Patients without Complex Lesions

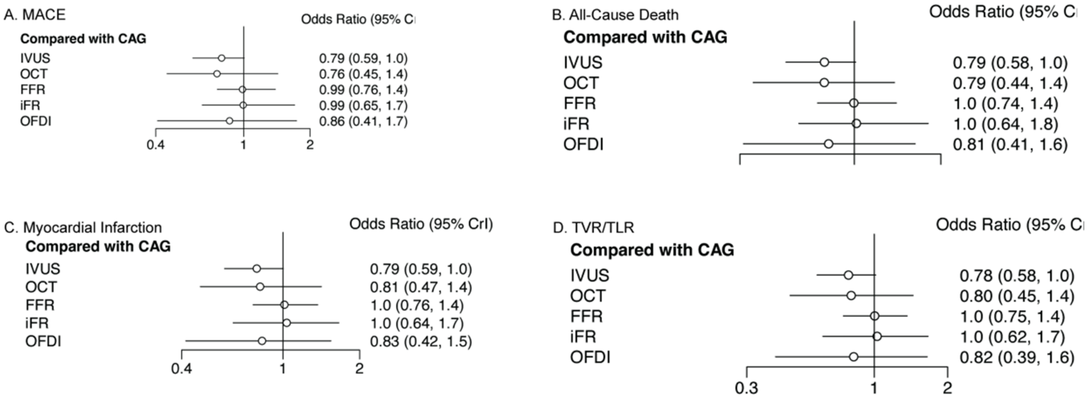

Figure S13 Comparisons of Stent Thrombosis Among Guidance Modalities Included in the Network Meta-Analysis

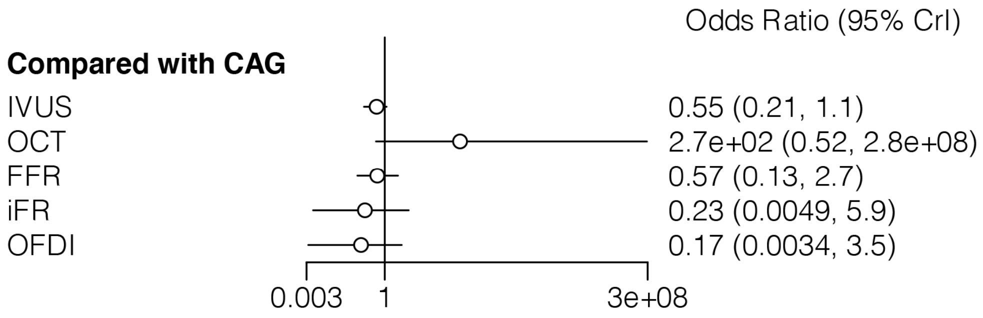

Supplement: Supplementary file 1 [file jcdd-09-00343-s001.zip › jcdd-1872392-Supplementary Figures.pdf]
