# Supplementary material for: Comparison of Six Different Percutaneous Coronary Intervention Guidance Modalities
Source: J Cardiovasc Dev Dis. 2022 Oct 8;9(10):343. doi: 10.3390/jcdd9100343 (PMC9604203; doi:10.3390/jcdd9100343)
Supplement: Supplementary file 1 [file jcdd-09-00343-s001.zip › jcdd-1872392-supplementary.pdf]

Table S1 Characteristics of Studies Included in the Network Meta-Analysis

Study/First author, year	Period	Center	Number of Patients	Stent Type	Inclusion criteria	Exclusion criteria	Primary outcome	MACE definition	Maximum follow-up
IVUS versus CAG									
SIPS,[1] 2000	1996.02-1996.05	single center	121/148	BMS	elective or urgent PTCA or primary stenting in vessels of diameter 2.2 and 4.6 mm	emergency intervention, planned atherectomy, CTO	minimal lumen diameter	death, MI, re-PTCA, and CABG	2 years
CRUISE,[2] 2000	1996.04-1997.05	multicenter	290/253	BMS	symptomatic ischemic heart disease, new lesions or restenotic lesions, and planned stent implantation	revascularization of lesions other than the stented lesion, aspirin, ticlopidine, or cumarin was contraindicated, LM disease, MI within 7 days, stroke/transient ischemic neurological attack within 3 months	minimal stent dimensions	death, MI, and TVR	9 months
OPTICUS,[3] 2001	1996.10-1998.02	multicenter	273/275	BMS	angina or ischemia, no	acute angina at rest, complete	restenosis rate, minimal	death, MI, CABG, and	1 year

					contraindication to antiplatelet therapy, and lesion length ≤25 mm	akinesia in the myocardium supplied by the target artery, significant LM lesion, bifurcation lesion, and involvement of a side branch ≥2 mm in diameter with ostial stenosis	lumen diameter, and percent diameter stenosis	repeat PCI	
Gaster,[4, 5] 2003	1997	single center	54/54	BMS	male patients referred for PCI	follow-up was unlikely, MI<3 months, unstable angina within a month, left bundle branch block, atrial fibrillation, serum creatinine >200 μmol/l, thyrotoxicosis, polycythemia, total occlusion	recurrence of stenosis	NA	2.5 years
TULIP,[6] 2003	1998.06-	single	74/76	BMS	nonostial	MI within 2 weeks,	cardiac	cardiac death,	1 year

	2001.01	center			stenosis ≥20 mm length with a reference diameter that permitted implantation of ≥3-mm stents without involvement of significant side branches (diameter ≥2.0 mm).	total occlusion, contraindications for combined antiplatelet therapy	death, MI, TLR	MI, TLR	
DIPOL,[7] 2007	2000.09-2002.12	multicenter	83/80	BMS	stable angina pectoris, age between 18 and 70 years, 1 or 2 “de novo” vessel disease, vessel reference diameter >2.75 mm, and lesion length ≥25 mm	recent MI or unstable angina, LM stenosis, large calcifications, large (>2 mm in diameter) side branch, and CTO	MACE	death, MI, and revascularization	6 months
AVID,[8] 2009	1995-	multicenter	394/406	BMS	>18 years,	dissection not	TLR	death, MI, stent	1 year

	1999				scheduled for elective coronary stent placement	covered by stent, TIMI <3 after stent placement, CTO, stent placement in a sole remaining circulation or LM equivalent, stent placement within an aneurysmal portion of a vessel, bypass graft supplying a native vessel <2.0 mm, cardiac transplantation, performance of IVUS during the index procedure before stent placement		thrombosis, CABG	
Jakabcin,[9] 2010	2004.01-2005.12	single center	105/105	DES	lesion type B2 and C, proximal LAD, LM disease, reference vessel	NA	MACE	death, MI, and revascularization	1.5 years

					diameter <2.5 mm, lesion length >20 mm, in-stent restenosis, insulin dependent diabetes mellitus, and ACS				
AVIO,[10] 2013	NA	multicenter	142/142	DES	complex lesions defined as one of the following: lesions >28 mm, CTO, lesions involving a bifurcation, small vessels (≤2.5mm), and requiring 4 or more stents	contraindication to dual antiplatelet therapy, LVEF <30%, renal failure, significant comorbidities precluding clinical follow-up, MI in the 48 hours prior to the procedure, in-stent restenosis, prior brachytherapy, venous or arterial grafts, unprotected	lesion minimal lumen diameter	MI, cardiac death, and TVR	2 years

						LM stenosis, thrombocytopenia <100,000, heart transplant, pregnant, acute infection, planned major surgery, DES implanted <1 year			
RESET,[11] 2013	2009.04-2010.12	multicenter	269/274	DES	lesion requiring a stent ≥28 mm	bleeding within 3 months, hypersensitivity to heparin, aspirin, clopidogrel, or a limus-related drug, cerebral vascular accident, peripheral artery occlusive diseases, thromboembolic disease, stent thrombosis, cardiogenic shock, LVEF <40%, STEMI within 48 h,	MACE	cardiac death, MI, TVR, or stent thrombosis	1 year

						LM disease, bifurcation lesions, CTO, and a history of PCI with DES.			
MOZART,[12] 2014	2012.11-2013.09	NA	41/42	NA	≥1 of the following criteria: 1) >75 years, diabetes, ACS needing urgent or emergent PCI, creatinine clearance <60 ml/min/1.73 m^2 or a single remaining kidney or previous renal transplantation, congestive heart failure, pulmonary congestion, LVEF <45%, cardiogenic shock, or intra-	use of iodinated contrast agents <72 h or other nephrotoxic agents <7 days, allergy to contrast agents, and unstable or unknown renal function before PCI	total volume contrast agent	NA	4 months

					aortic balloon pumping				
Tan[13], 2015	2009.10-2012.09	single center	61/62	DES	>70 years, LM disease	LVEF <30%, cardiogenic shock, MI, carcinoma.	MACE	death, MI, and TLR	2 years
AIR-CTO[14], 2015	2010.10-2011.11	multicenter	115/115	DES	18-80 years, silent ischaemia, stable angina, unstable angina or previous MI, CTO	age >80 years, pregnant, liver dysfunction, creatinine >2.5 mg/dl, major bleeding or stroke within six months, platelet count <8×10^9/L, white blood cells <40×10^9/L, life expectancy <12 months, allergy to the study medications, failure of recanalisation in a CTO lesion, STEMI <24 hours, and intolerance to	in-stent late lumen loss	NA	2 years

						dual antiplatelet therapy			
IVUS-XPL,[15] 2015	2010.10-2014.07	multicenter	700/700	DES	chest pain or MI, long coronary lesions (implanted stent ≥28 mm)	MI within 48 h, contraindication for anti-platelet agents and bleeding within 3 months, cerebral vascular accident, peripheral artery occlusive disease, thromboembolic disease, stent thrombosis, >80 years, severe hepatic dysfunction, significant renal dysfunction, significant leucopenia, neutropenia, thrombocytopenia, anemia, or known bleeding diathesis,	MACE	cardiac death, MI, and TVR	1 year

						cardiogenic shock, LVEF <40%, pregnant, life expectancy <1 year, LM disease requiring PCI, bifurcation lesion, CTO, implanting DES within 6 months, in-stent restenosis			
CTO-IVUS,[16] 2015	2012.03-2013.08	multicenter	201/201	DES	CTO, 20 to 80 years, typical symptomatic angina or ischemia	LM disease, in-stent restenosis, ACS, LVEF <30%, IVUS use before randomization	cardiac death	cardiac death, MI, and TVR	1 year
Wang,[17] 2015	2012.10-2013.08	single center	38/42	DES	STEMI within 12 h, preprocedural TIMI grade 0/1 flow or thrombus grade ≥3 in the IRA, 50–75% residual stenosis after aspiration	residual stenosis >75% or TIMI grade <3 flow after aspiration thrombectomy, ≥2 stents inserted, LM occlusion, hemodynamic	MACE	cardiac death, MI, TVR, and intractable myocardial ischemia	1 year

					thrombectomy and a TIMI grade 3 flow at the distal end of the IRA	instability, old MI, prior cardiopulmonary resuscitation, hepatic and renal dysfunction or neoplastic disease, valvular heart disease, congenital heart disease, or cardiomyopathy, coronary angioplasty or CABG, coagulation disorders, no tolerance for aspirin, clopidogrel, heparin and contrast medium allergies			
ULTIMATE,[18] 2018	2014.08-2017.05	multicenter	724/724	DES	silent ischemia, stable or unstable angina, MI >24 h, and a	life expectancy <12 months, intolerant of antithrombotic therapy, significant	target-vessel failure	cardiac death, MI, and TVR	1 year

					de novo coronary lesion eligible for DES implantation	anemia, thrombocytopenia, or leucopenia, history of major hemorrhage, CTO, severe calcification needing rotational atherectomy			
SURF,[19] 2019	2012.11-2017.11	single center	688/700	DES	>18 years referred for CAG and PCI	cardiogenic shock, dialysis, severe peripheral vascular disease, previous failed access, or a failed Allen's test	major bleeding, MACE	death, stroke, MI or urgent TLR	30 days
OCT versus CAG									
DOCTORS,[20] 2016	2013.09-2015.12	multicenter	120/120	DES	18 to 80 years, NSTEMI	LM disease, in-stent restenosis, presence of coronary artery bypass grafts, cardiogenic shock or severe hemodynamic instability, severely	FFR	NA	6 months

						calcified or tortuous arteries, persistent ST-segment elevation, ≥1 lesions considered angiographically significant, or nonsignificant diffuse disease, severe renal insufficiency, bacteremia or septicemia, severe coagulation disorders; pregnancy			
ROBUST,[21] 2018	2011.02-2012.10	multicenter	105/96	DES	18 and 85 years, STEMI	cardiogenic shock, LM disease, and ostial lesion	MACE	death, MI, and TLR	9 months
OPTICO BVS,[22] 2020	2016.06-2017.05	multicenter	19/19	BVS	≥18 years, stable angina, or ACS	LM disease, ostial lesion, severe calcified lesions, and bifurcation with sidebranch >2.5	in-scaffold minimal lumen area	NA	6 months

						mm or any sidebranch that possibly requires treatment			
OFDI versus CAG									
OPTIMUM,[23] 2020	2017.06-2018.09	multicenter	56/54	DES	>18 years, CAD involving a bifurcation with objective evidence of ischemia, bifurcation lesion, side branch >2.0mm	pregnancy, intolerance to aspirin, clopidogrel, heparin, cobalt chromium, sirolimus, contrast material, thrombocytopaenia, Contraindications to PCI, stenting, clopidogrel, prasugrel or ticagrelor, cardiogenic shock, planned surgery, history of stenting in the target bifurcation lesion, renal insufficiency,	percentage of malapposed struts	NA	1 year

						severely tortuous or angulated coronary anatomy			
OCT versus IVUS									
Habara,[24] 2012	2010.03-2010.11	single center	35/35	BMS DES	symptomatic ischemic heart disease, de novo lesion in the native coronary circulation, and planned stent implantation	LM disease, totally occluded lesion, diffuse lesion, bifurcation lesion, lesion of large vessel, severe tortuous vessel, cardiogenic shock, LVEF <30%, serum creatinine >2 mg/dL, STEMI, and contraindication to aspirin, ticlopidine, or heparin	stent expansion	NA	in-hospital
FFR versus CAG									
FAME,[25-27] 2015	2006.01-2007.09	multicenter	509/496	DES	MVD	MI within 5 days, LM disease, previous CABG, cardiogenic shock, extremely tortuous	MACE	death, MI, and revascularization	5 years

						or calcified coronary arteries, life expectancy <2 years, contraindication to DES, and pregnant			
DEFER-DES[28], 2015	2006.11-2007.12	multicenter	114/115	DES	intermediate stenosis (40%–70% diameter stenosis)	LM disease, cardiogenic shock, chronic kidney disease, life expectancy <2 years, conduction disturbance more than first-degree AV block, and contraindication to adenosine	MACE	cardiac death, MI, and TLR	5 years
FAMOUS–NSTEMI,[29] 2015	2011.10-2013.05	multicenter	176/174	BMS DES	recent NSTEMI and with at least one risk factor for CAD	ischaemic symptoms that were not controlled by medical therapy, haemodynamic instability, STEMI, intolerance to anti-	proportion of patients allocated to medical management	cardiac death, MI, and heart failure	1 year

						platelet drugs, ineligible for coronary revascularization, a treatment plan for non-coronary heart surgery, prior CABG, severe or mild coronary disease, life expectancy <1 year			
DKCRUSH-VI,[30] 2015	2011.11-2013.06	multicenter	160/160	DES	silent ischemia, stable or unstable angina with a single true coronary bifurcation lesion, diameter of stenosis ≥50%	MI within 1 month, LVEF <30%, previous CABG, target vessel stenting or stroke within 6 months, distal LM trifurcation with non-recanalized RCA CTO, calcification, planned surgery necessitating	MACE	cardiac death, MI, and TVR	1 year

						antiplatelet therapy interruption within 6 months post-PCI, drug contraindication or intolerance, glomerular filtration rate <40 ml/min/ 1.73 m^2, platelet count $<10x10^9$/L, liver dysfunction, pregnancy, expected life span <1 year			
Zhang,[31] 2016	2013.09-2016.06	single center	110/110	DES	> 65 years, NSTEMI	ongoing ischemic symptoms, cardiogenic shock or hemodynamic instability, intolerance to antiplatelet drugs, ineligibility for PCI, stenosis <30% severity, highly	MACE	cardiovascular death, MI, and heart failure	1 year

						tortuous or calcified coronary arteries, noncoronary cardiosurgery, and life expectancy <1 year			
Quintella,[32] 2019	2011.04-2016.05	single center	34/35	DES	>21 years, MVD, at day 7 after ACS	severe left ventricular dysfunction	MACE	death, angina, and restenosis	< 12 months
FLOWER-MI,[33] 2021	2016.12-2018.12	multicenter	586/577	DES	≥18 years, STEMI, MVD, successful PCI of IRA	single-vessel disease, hemodynamic instability, previous CABG, coronary-artery calcification or CTO, failed culprit-lesion PCI, and referral for CABG	MACE	death, nonfatal MI, or unplanned hospitalization leading to urgent revascularization	1 year
OFDI versus IVUS									
OPINION,[34] 2017	2013.06-2014.07	multicenter	412/405	DES	20-85 years scheduled for PCI	MI within 3 months, cardiogenic shock, congestive heart	target vessel failure	cardiac death, MI, and TLR	1 year

						failure, chronic kidney disease, haemodialysis or peritoneal dialysis, planned surgery within 1 year after PCI, scheduled PCI with BMS, triple-vessel disease, LM disease, aorto-ostial lesion, CTO, small vessel disease, CABG, in-stent restenosis			
MISTIC-1,[35] 2020	2014.06-2016.08	multicenter	54/55	DES	>20 years, stable CAD	renal insufficiency, LVEF <30%, congestive heart failure, ACS within 7 days, nonsuitability for DES use or dual antiplatelet therapy, life expectancy <1 year, lesion	in-segment minimum lumen area	cardiovascular mortality, MI, or TLR	3 years

						length >28 mm, CTO, LM lesion, bifurcation lesion, orseverely calcified lesion			
OCT versus FFR									
FORZA,[36] 2020	2013.03-2018.05	single center	174/176	DES	>18 years, ischemic heart disease, SVD with intermediate coronary lesion, MVD with only intermediate coronary lesion, MVD with ≥1 intermediate coronary lesion and already treated angiographically critical stenosis	pregnant, life expectancy <12 months, follow up difficult, LVEF ≤30%, STEMI <7 days, NSTEMI <48 hours, prior STEMI in the territory supplied by the vessel with the intermediate under investigation, severe myocardial hypertrophy, severe valvular heart disease, significant platelet count alteration,	significant residual angina	death, MI, and TVR	13 months

						gastrointestinal bleeding, clotting pathology, hypersensitivity to aspirin, heparin, or contrast dye, glomerular filtration rate <30 ml/min, MVD with ≥1 untreated angiographically critical stenosis or coronary occlusion, lesions in coronary artery bypass grafts, and MVD requiring CABG			
FFR versus iFR									
DEFINE-FLAIR,[37] 2017	2014.01-2015.12	multicenter	1242/1250	DES	>18 years, eligible for PCI, CAD, ≥1 native artery with physiological severity, stable	previous CABG, LM disease, tandem stenoses, CTO, restenotic lesions, haemodynamic	MACE	death, MI, and revascularization	1 year

					angina or ACS	instability, contraindication to adenosine administration, PCI, or DES, calcified or tortuous vessels, hepatic or lung disease, pregnancy, STEMI within 48 hours, valvular heart disease, ≥ 1 target vessel			
iFR-SWEDEHEART,[38] 2017	2014.05-2015.10	multicenter	1019/1018	DES	≥ 18 years, stable angina pectoris or unstable angina pectoris/NSTEMI	previous CABG with patent grafts to the interrogated vessel, life expectancy <1 year, MVD, Killip class III-IV, inability to tolerate adenosine, calcified or tortuous vessels	MACE	death, MI, and revascularization	1 year

OCT versus IVUS versus CAG									
ILUMIEN III,[39, 40] 2021	2015.05-2016.04	multicenter	158/146/146	DES	>18 years, angina, silent ischaemia, NSTEMI, recent STEMI >24 h from initial presentation, vessel diameter of 2.25–3.50 mm, length < 40 mm	LM or ostial RCA stenoses, bypass graft stenoses, CTO, planned two-stent bifurcations, and in-stent restenosis	post-PCI minimum stent area	death, MI, stent thrombosis, or repeat revascularisation	1 year

ACS: acute coronary syndrome; BMS: bare metal stent; BVS: bioresorbable vascular scaffolds; CABG: coronary artery bypass graft; CAD: coronary artery disease; CAG: coronary angiography; CTO: chronic total occlusion; DES: drug-eluting stents; FFR: fractional flow reserve; iFR: instantaneous wave-free ratio; IRA: infarct related artery; IVUS: intravenous ultrasound; LAD: left anterior descending artery; LM: left main coronary artery; LVEF: left ventricular ejection fractions; MACE: major adverse cardiovascular events; MI: myocardial infarction; MVD: multivessel coronary artery disease; NSTEMI: non-ST segment elevation myocardial infarction; PCI: percutaneous coronary intervention; OCT: optical coherence tomography; OFDI: optical frequency domain imaging; PTCA: percutaneous transluminal coronary angioplasty; RCA: right coronary artery; STEMI: ST elevation myocardial infarction; SVD: single vessel disease; TIMI: thrombolysis in myocardial infarction; TLR: target lesion revascularization; TVR: target vessel revascularization

Table S2 Clinical Characteristics of Patients Across Studies Included in the Network Meta-Analysis

Study/First author, year	Age	Male, %	Diabetes, %	Hypertension, %	Dyslipidemia, %	Smoker, %	Previous MI, %	Previous PCI, %	Previous CABG, %	ACS, %	LVEF, %	LM, %	LAD, %	LCX, %	RCA, %
IVUS versus CAG															
SIPS,[1] 2000	61.2±8.1/60.7±9.6	82/76	16/16	64/56	88/87	47/45	58/52	NA	11/10	NA	NA	0	38/41	27/27	30/30
CRUISE,[2] 2000	60±11/ 61±11	69/72	23/18	52/59	39/33	NA	32/41	NA	9/7	0	55±10 54±12	0	43/46	24/18	33/36
OPTICUS,[3] 2001	60.1±10.0/61.5±9.5	77/78	17/17	48/52	61/67	69/66	32/32	20/20	3/4	13/12	56.5±14.0/57.7±14.3	0	51/50	18/14	30/35
Gaster,[4, 5] 2003	57/57	NA	4/11	20/24	96/93	30/15	54/44	6/15	7/4	0	NA	NA	NA	NA	NA
TULIP,[6] 2003	61±10/63±10	71/72	16/21	27/30	61/62	40/53	NA	NA	NA	0	<40% (0)	0	39/38	10/21	51/41
DIPOL,[7] 2007	56±8/54±8	81/83	10/11	NA	47/40	47/52	44/40	NA	NA	0	52±9/48±10	0	41/46	26/24	33/30
AVID,[8] 2009	62±12/63±11	73/68	15/17	46/45	40/44	NA	35/29	24/25	18/20	0	53±13/55±13	0.8/0.5	40/37	15/18	35/32
Jakabcin,[9] 2010	59.4±13/60.2±11	73/71	42/45	67/71	63/66	40/35	37/32	17/14	14/10	62/60	NA	3/4	56/54	11/15	29/24
AVIO,[10] 2013	63.9±10.1/63.6±11.0	82.4/76.8	23.9/26.8	70.4/66.9	70.4/76.8	34.5/31.0	NA	NA	NA	0	55.3±8.5/55.9±8.6	0	53.3/ 48.6	NA	NA
RESET,[11] 2013	62.8±9.3/64.3±8.7	65.8/54.7	31.6/29.9	61.3/65.8	61.3/61.7	21.6/17.2	1.1/2.9	NA	NA	8.9/9/9	55.3±23.9/54.0±25.0	0	50.0/57.5	20.7/18.4	29.3/24.1
MOZART,[12] 2014	67.1/62.1	61.0/57.	73.2/81.0	97.6/100	NA	41.5/40.4	NA	26.8/11.9	14.6/16.7	14.6/16.	NA	4.9/7.1	34.1/52.	46.3/	22.0/

		1								7			4	28.6	35.7
Tan[13], 2015	76.54±4.95/ 75.85±3.49	62.3/69.4	34.4/ 29.5	41.0/ 46.8	NA	44.3/ 46.8	16.4/ 21.0	NA	NA	70.5/66.1	55.32±5.02/ 53.33±7.14	100/100	0	0	0
AIR-CTO[14], 2015	67/66	88.7/80.0	29.6/27.0	74.8/70.4	21.7/27.8	39.1/39.1	NA	NA	NA	28.7/24.4	55/56	0/2.6	44.3/36.5	20.9/14.8	34.8/46.1
IVUS-XPL,[15] 2015	64±9/64±9	69/69	36/37	65/63	67/65	22/26	5/4	11/10	3/2	14/17	62.9±9.8/62.4±10.2	0	65/60	14/15	21/25
CTO-IVUS,[16] 2015	61.0±11.1/ 61.4±10.1	80.6/ 80.6	34.8/ 33.8	62.7/ 63.7	NA	35.3/ 34.3	8.0/ 8.0	15.4/ 15.9	1.5/.5	0	56.9±13.1/ 56.7±11.4	0	41.8/ 46.8	14.4/ 15.9	43.8/ 37.3
Wang,[17] 2015	56.4±9.4/ 53.7±11.8	60.5/66.7	21.1/11.9	39.5/23.8	26.3/23.8	50.0/59.5	NA	NA	0	100/100	48.3±5.7/49. ±5.9	0	39.5/42.9	15.8/7.1	44.7/50.0
ULTIMATE,[18] 2018	65.2±10.9/65.9±9.8	73.9/73.2	30.0/31.2	70.7/72.0	53.7/55.2	34.9/31.5	9.3/11.9	17.4/19.9	1.4/1.1	11.2/14.0	60.9±7.9/60.3±9.3	9.9/8.6	47.5/46.7	17.3/16.8	25.4/28.0
SURF,[19] 2019	63.38±11.1/63.60±11.5	70.9/74.0	32.8/35.1	64.5/66.7	63.1/60.7	61.2/62.3	14.7/16.4	20.5/20.0	3.3/4.3	76.5/73.8	NA	NA	NA	NA	NA
OCT versus CAG															
DOCTORS,[20] 2016	60.8±11.5/ 60.2±11.3	79.2/ 75.8	21.7/ 15.8	55.8/ 41.7	49.2/ 46.7	39.2/ 42.5	NA	NA	0	100/100	NA	0	NA	NA	NA
ROBUST,[21] 2018	57/59	83/87	17/26	50/52	NA	64/59	1/6	4/4	0	100/100	NA	0	39/32	16/12	48/52
OPTICO BVS,[22] 2020	63.3±12.7/ 62.9±9.1	79/79	21/21	37/58	68/63	37/32	21/11	32/16	NA	53/26	61.0±7.6/ 64.4±10.5	0	38/40	24/10	38/50

OFDI versus CAG															
OPTIMUM,[23] 2020	68.9±10.2/ 69.4±11.6	79/74	51.8/46.3	76.8/74.1	85.7/85.2	23.2/18.5	16.1/14.8	3.6/0	21.4/35.2	8.9/5.6	60.8±14.3/ 59.7±11.8	1.8/14.8	73.7/51.9	15.8/14.8	8.8/18.5
OCT versus IVUS															
Habara,[24] 2012	67.6±9.7/67.4±8.0	82.9/74.3	31.4/25.7	31.4/25.7	42.9/60	20/8.6	8.6/8.6	17.1/11.4	2.9/2.9	11.4/8.6	NA	0	40/48.6	22.9/31.4	37.1/20
FFR versus CAG															
FAME,[25-27] 2015	64.6±10.3/ 64.2±10.2	75.4/ 72.6	24.2/25.2	61.3/65.9	71.9/73.0	27.1/31.5	36.7/36.3	28.7/26.0	0	29.4/35.8	57.2±11.0/ 57.1±12.0	0	NA	NA	NA
DEFER-DES[28], 2015	62±10/ 63±10	73/75	26/34	64/57	70/68	26/33	19/17	19/17	NA	51/48	62±9/61±9	0	55/56	21/15	27/30
FAMOUS–NSTEMI,[29] 2015	62.3±11.0/ 61.6±11.1	75.6/73.0	14.8/14.9	44.3/46.6	40.3/32.2	40.9/40.8	12.5/13.8	10.8/10.9	0	100/100	NA	NA	NA	NA	NA
DKCRUSH-VI,[30] 2015	65.2±9.6/65.4±9.2	75.6/72.5	30.0/26.9	72.5/68.3	16.9/20.0	41.3/40.0	7.5/11.9	16.3/11.9	0	61.7/61.9	61.3±7.4/60.6±8.7	9.4/8.8	68.2/71.3	18.1/14.3	4.4/5.6
Zhang,[31] 2016	70±3.7/ 70±3.4	68.2/ 70.9	36.4/ 32.7	73.6/ 75.5	81.8/ 84.5	26.4/ 28.2	21.8/ 20.9	NA	NA	100/100	NA	NA	NA	NA	NA
Quintella,[32] 2019	62.7±8.4/ 59.5±9.4	53.2/ 46.8	50.0/ 50.0	49.0/ 50.9	42.0/ 52.0	52.6/ 47.4	53.3/ 46.7	NA	NA	57.1/ 42.8	70.0±14.0/64.0±12.0	NA	NA	NA	NA
FLOWER-MI,[33] 2021	62.5±11.0/ 61.9±11.4	85.0/ 81.1	18.3/14.2	43.2/45.4	39.6/41.1	40.1/36.4	7.7/5.4	10.1/7.6	0	100/100	50/50	0.5/0.7	31.1/34.0	21.2/22.0	48.0/45.1

OFDI versus IVUS															
OPINION,[34] 2017	69±9/ 68±9	76.5/79.5	41.0/ 40.7	76.5/73.8	76.7/ 79.3	16.3/ 18.0	17.0/15.1	34.0/34.6	1.7/ 2.2	13.1/11.7	NA	0	54.1/48.6	20.4/21.5	24.8/28.9
MISTIC-1,[35] 2020	72/71	75.9/80.0	50.0/43.6	63.0/70.9	79.6/65.5	40.7/21.8	35.2/29.1	44.4/47.3	0	0	58/57	0	50.0/39.1	21.0/26.6	29.0/34.4
OCT versus FFR															
FORZA,[36] 2020	69±9/ 68±10	77.6/71.6	36.2/34.7	86.8/84.1	84.7/68.2	37.9/39.8	29.9/18.8	43.7/41.5	2.9/2.3	17.8/21.0	56±9/60±8	0	60.6/66.7	12.2/16.4	27.1/16.9
FFR versus iFR															
DEFINE-FLAIR,[37] 2017	65.5±10.8/ 65.2±10.6	77.5/74.3	30.8/30.1	70.3/70.7	63.9/63.4	56.7/56.4	28.8/30.1	39.4/42.2	0	15.0/14.7	NA	0	53.6/52.5	20.5/20.7	23.7/24.4
iFR-SWEDEHEART,[38] 2017	67.6±9.6/67.4±9.2	74.2/75.2	22.8/20.9	71.6/69.7	71.9/69.2	64.8/62.2	33.1/32.9	42.1/41.7	4.8/4.2	17.3/17.5	NA	1.5/1.6	47.4/47.9	19.2/18.3	17.9/20.0
OCT versus IVUS versus CAG															
ILUMIEN III,[39, 40] 2021	66/66/67	69/73/73	33/38/29	78/77	75	18/13/24	22/20/22	7/5/10	2/8/5	13/13/16	NA	0	51/47/57	27/29/21	22/25/22

Values refer to corresponding treatment arms from original studies.

ACS: acute coronary syndrome; CABG: coronary artery bypass graft; CAG: coronary angiography; FFR: fractional flow reserve; iFR: instantaneous wave-free ratio; IVUS: intravenous ultrasound; LAD: left anterior descending artery; LCX: left circumflex artery; LM: left main coronary artery; LVEF: left ventricular ejection fractions; NA: not available; MI: myocardial infarction; OCT: optical coherence tomography; OFDI: optical frequency domain imaging; PCI: percutaneous coronary intervention; RCA: right coronary artery

References

1. Frey AW, Hodgson JM, Müller C, Bestehorn HP, Roskamm H. Ultrasound-guided strategy for provisional stenting with focal balloon combination catheter: results from the randomized Strategy for Intracoronary Ultrasound-guided PTCA and Stenting (SIPS) trial. Circulation 2000;102(20):2497-502.
2. Fitzgerald PJ, Oshima A, Hayase M, Metz JA, Bailey SR, Baim DS, et al. Final results of the Can Routine Ultrasound Influence Stent Expansion (CRUISE) study. Circulation 2000;102(5):523-30.
3. Mudra H, di Mario C, de Jaegere P, Figulla HR, Macaya C, Zahn R, et al. Randomized comparison of coronary stent implantation under ultrasound or angiographic guidance to reduce stent restenosis (OPTICUS Study). Circulation 2001;104(12):1343-9.
4. Gaster AL, Slothuus U, Larsen J, Thayssen P, Haghfelt T. Cost-effectiveness analysis of intravascular ultrasound guided percutaneous coronary intervention versus conventional percutaneous coronary intervention. Scand Cardiovasc J 2001;35(2):80-5.
5. Gaster AL, Slothuus Skjoldborg U, Larsen J, Korsholm L, von Birgelen C, Jensen S, et al. Continued improvement of clinical outcome and cost effectiveness following intravascular ultrasound guided PCI: insights from a prospective, randomised study. Heart 2003;89(9):1043-9.
6. Oemrawsingh PV, Mintz GS, Schalij MJ, Zwinderman AH, Jukema JW, van der Wall EE. Intravascular ultrasound guidance improves angiographic and clinical outcome of stent implantation for long coronary artery stenoses: final results of a randomized comparison with angiographic guidance (TULIP

Study). Circulation 2003;107(1):62-7.

7. Gil RJ, Pawłowski T, Dudek D, Horszczaruk G, Zmudka K, Lesiak M, et al. Comparison of angiographically guided direct stenting technique with direct stenting and optimal balloon angioplasty guided with intravascular ultrasound. The multicenter, randomized trial results. Am Heart J 2007;154(4):669-75.

8. Russo RJ, Silva PD, Teirstein PS, Attubato MJ, Davidson CJ, DeFranco AC, et al. A randomized controlled trial of angiography versus intravascular ultrasound-directed bare-metal coronary stent placement (the AVID Trial). Circ Cardiovasc Interv 2009;2(2):113-23.

9. Jakabcin J, Spacek R, Bystron M, Kvasnák M, Jager J, Veselka J, et al. Long-term health outcome and mortality evaluation after invasive coronary treatment using drug eluting stents with or without the IVUS guidance. Randomized control trial. HOME DES IVUS. Catheter Cardiovasc Interv 2010;75(4):578-83.

10. Chieffo A, Latib A, Caussin C, Presbitero P, Galli S, Menozzi A, et al. A prospective, randomized trial of intravascular-ultrasound guided compared to angiography guided stent implantation in complex coronary lesions: the AVIO trial. Am Heart J 2013;165(1):65-72.

11. Kim JS, Kang TS, Mintz GS, Park BE, Shin DH, Kim BK, et al. Randomized comparison of clinical outcomes between intravascular ultrasound and angiography-guided drug-eluting stent implantation for long coronary artery stenoses. JACC Cardiovasc Interv 2013;6(4):369-76.

12. Mariani J, Jr., Guedes C, Soares P, Zalc S, Campos CM, Lopes AC, et al. Intravascular ultrasound guidance to minimize the use of iodine contrast in percutaneous coronary intervention: the MOZART (Minimizing cOntrast utiliZation With IVUS Guidance in coRonary angioplasTy) randomized controlled trial. JACC Cardiovasc Interv 2014;7(11):1287-93.

13. Tan Q, Wang Q, Liu D, Zhang S, Zhang Y, Li Y. Intravascular ultrasound-guided unprotected left main coronary artery stenting in the elderly. Saudi Med J 2015;36(5):549-53.

14. Tian NL, Gami SK, Ye F, Zhang JJ, Liu ZZ, Lin S, et al. Angiographic and clinical comparisons of intravascular ultrasound- versus angiography-guided drug-eluting stent implantation for patients with chronic total occlusion lesions: two-year results from a randomised AIR-CTO study. EuroIntervention 2015;10(12):1409-17.

15. Hong SJ, Kim BK, Shin DH, Nam CM, Kim JS, Ko YG, et al. Effect of Intravascular Ultrasound-Guided vs Angiography-Guided Everolimus-Eluting Stent Implantation: The IVUS-XPL Randomized Clinical Trial. Jama 2015;314(20):2155-63.

16. Kim BK, Shin DH, Hong MK, Park HS, Rha SW, Mintz GS, et al. Clinical Impact of Intravascular Ultrasound-Guided Chronic Total Occlusion Intervention With Zotarolimus-Eluting Versus Biolimus-Eluting Stent Implantation: Randomized Study. Circ Cardiovasc Interv 2015;8(7):e002592.

17. Wang HX, Dong PS, Li ZJ, Wang HL, Wang K, Liu XY. Application of Intravascular Ultrasound in the Emergency Diagnosis and Treatment of Patients

with ST-Segment Elevation Myocardial Infarction. Echocardiography 2015;32(6):1003-8.

18. Zhang J, Gao X, Kan J, Ge Z, Han L, Lu S, et al. Intravascular Ultrasound Versus Angiography-Guided Drug-Eluting Stent Implantation: The ULTIMATE Trial. J Am Coll Cardiol 2018;72(24):3126-3137.

19. Nguyen P, Makris A, Hennessy A, Jayanti S, Wang A, Park K, et al. Standard versus ultrasound-guided radial and femoral access in coronary angiography and intervention (SURF): a randomised controlled trial. EuroIntervention 2019;15(6):e522-e530.

20. Meneveau N, Souteyrand G, Motreff P, Caussin C, Amabile N, Ohlmann P, et al. Optical Coherence Tomography to Optimize Results of Percutaneous Coronary Intervention in Patients with Non-ST-Elevation Acute Coronary Syndrome: Results of the Multicenter, Randomized DOCTORS Study (Does Optical Coherence Tomography Optimize Results of Stenting). Circulation 2016;134(13):906-17.

21. Kala P, Cervinka P, Jakl M, Kanovsky J, Kupec A, Spacek R, et al. OCT guidance during stent implantation in primary PCI: A randomized multicenter study with nine months of optical coherence tomography follow-up. Int J Cardiol 2018;250:98-103.

22. Ueki Y, Yamaji K, Barbato E, Nef H, Brugaletta S, Alfonso F, et al. Randomized Comparison of Optical Coherence Tomography Versus Angiography to Guide Bioresorbable Vascular Scaffold Implantation: The OPTICO BVS Study. Cardiovasc Revasc Med 2020;21(10):1244-1250.

23. Onuma Y, Kogame N, Sotomi Y, Miyazaki Y, Asano T, Takahashi K, et al. A Randomized Trial Evaluating Online 3-Dimensional Optical Frequency

Domain Imaging-Guided Percutaneous Coronary Intervention in Bifurcation Lesions. Circ Cardiovasc Interv 2020;13(12):e009183.

24. Habara M, Nasu K, Terashima M, Kaneda H, Yokota D, Ko E, et al. Impact of frequency-domain optical coherence tomography guidance for optimal coronary stent implantation in comparison with intravascular ultrasound guidance. Circ Cardiovasc Interv 2012;5(2):193-201.

25. Tonino PA, De Bruyne B, Pijls NH, Siebert U, Ikeno F, van' t Veer M, et al. Fractional flow reserve versus angiography for guiding percutaneous coronary intervention. N Engl J Med 2009;360(3):213-24.

26. Pijls NH, Fearon WF, Tonino PA, Siebert U, Ikeno F, Bornschein B, et al. Fractional flow reserve versus angiography for guiding percutaneous coronary intervention in patients with multivessel coronary artery disease: 2-year follow-up of the FAME (Fractional Flow Reserve Versus Angiography for Multivessel Evaluation) study. J Am Coll Cardiol 2010;56(3):177-84.

27. van Nunen LX, Zimmermann FM, Tonino PA, Barbato E, Baumbach A, Engstrøm T, et al. Fractional flow reserve versus angiography for guidance of PCI in patients with multivessel coronary artery disease (FAME): 5-year follow-up of a randomised controlled trial. Lancet 2015;386(10006):1853-60.

28. Park SH, Jeon KH, Lee JM, Nam CW, Doh JH, Lee BK, et al. Long-Term Clinical Outcomes of Fractional Flow Reserve-Guided Versus Routine Drug-Eluting Stent Implantation in Patients With Intermediate Coronary Stenosis: Five-Year Clinical Outcomes of DEFER-DES Trial. Circ Cardiovasc Interv 2015;8(12):e002442.

29. Layland J, Oldroyd KG, Curzen N, Sood A, Balachandran K, Das R, et al. Fractional flow reserve vs. angiography in guiding management to optimize outcomes in non-ST-segment elevation myocardial infarction: the British Heart Foundation FAMOUS-NSTEMI randomized trial. Eur Heart J 2015;36(2):100-11.

30. Chen SL, Ye F, Zhang JJ, Xu T, Tian NL, Liu ZZ, et al. Randomized Comparison of FFR-Guided and Angiography-Guided Provisional Stenting of True Coronary Bifurcation Lesions: The DKCRUSH-VI Trial (Double Kissing Crush Versus Provisional Stenting Technique for Treatment of Coronary Bifurcation Lesions VI). JACC Cardiovasc Interv 2015;8(4):536-46.

31. Zhang Z, Li K, Tian J. Efficacy and safety outcomes of fractional flow reserve in guiding clinical therapy of non-ST-segment elevation myocardial infarction compared with angiography alone in elderly Chinese patients. Clin Interv Aging 2016;11:1751-1754.

32. Quintella EF, Ferreira E, Azevedo VMP, Araujo DV, Sant'Anna FM, Amorim B, et al. Clinical Outcomes and Cost-Effectiveness Analysis of FFR Compared with Angiography in Multivessel Disease Patient. Arq Bras Cardiol 2019;112(1):40-47.

33. Puymirat E, Cayla G, Simon T, Steg PG, Montalescot G, Durand-Zaleski I, et al. Multivessel PCI Guided by FFR or Angiography for Myocardial Infarction. N Engl J Med 2021;385(4):297-308.

34. Kubo T, Shinke T, Okamura T, Hibi K, Nakazawa G, Morino Y, et al. Optical frequency domain imaging vs. intravascular ultrasound in percutaneous

coronary intervention (OPINION trial): one-year angiographic and clinical results. Eur Heart J 2017;38(42):3139-3147.

35. Muramatsu T, Ozaki Y, Nanasato M, Ishikawa M, Nagasaka R, Ohota M, et al. Comparison Between Optical Frequency Domain Imaging and Intravascular Ultrasound for Percutaneous Coronary Intervention Guidance in Biolimus A9-Eluting Stent Implantation: A Randomized MISTIC-1 Non-Inferiority Trial. Circ Cardiovasc Interv 2020;13(11):e009314.

36. Burzotta F, Leone AM, Aurigemma C, Zambrano A, Zimbardo G, Arioti M, et al. Fractional Flow Reserve or Optical Coherence Tomography to Guide Management of Angiographically Intermediate Coronary Stenosis: A Single-Center Trial. JACC Cardiovasc Interv 2020;13(1):49-58.

37. Davies JE, Sen S, Dehbi HM, Al-Lamee R, Petraco R, Nijjer SS, et al. Use of the Instantaneous Wave-free Ratio or Fractional Flow Reserve in PCI. N Engl J Med 2017;376(19):1824-1834.

38. Götberg M, Christiansen EH, Gudmundsdottir IJ, Sandhall L, Danielewicz M, Jakobsen L, et al. Instantaneous Wave-free Ratio versus Fractional Flow Reserve to Guide PCI. N Engl J Med 2017;376(19):1813-1823.

39. Ali ZA, Maehara A, Généreux P, Shlofmitz RA, Fabbiocchi F, Nazif TM, et al. Optical coherence tomography compared with intravascular ultrasound and with angiography to guide coronary stent implantation (ILUMIEN III: OPTIMIZE PCI): a randomised controlled trial. Lancet 2016;388(10060):2618-2628.

40. Ali ZA, Karimi Galougahi K, Maehara A, Shlofmitz RA, Fabbiocchi F, Guagliumi G, et al. Outcomes of optical coherence tomography compared with intravascular ultrasound and with angiography to guide coronary stent implantation: one-year results from the ILUMIEN III: OPTIMIZE PCI trial. EuroIntervention 2021;16(13):1085-1091.
